# Understanding Scaling Development in Intermittent MD Operation

**DOI:** 10.3390/membranes16040144

**Published:** 2026-04-09

**Authors:** Yair Morales, Jan Singer, Leonardo Acero, Harald Horn, Florencia Saravia

**Affiliations:** 1 DVGW-Research Center at the Engler-Bunte-Institut, Water Chemistry and Water Technology, Karlsruhe Institute of Technology, Engler-Bunte-Ring 9, 76131 Karlsruhe, Germany; 2 Engler-Bunte-Institut, Water Chemistry and Water Technology, Karlsruhe Institute of Technology, Engler-Bunte-Ring 9, 76131 Karlsruhe, Germany

**Keywords:** membrane distillation, desalination, scaling, fouling, operation

## Abstract

Membrane distillation (MD) is an attractive technology for desalination driven by renewable energy and low-grade heat sources. However, specific practical guidelines for intermittent operations, typical of such alternative energy sources, are still limited—particularly with respect to established shutdown measures to mitigate adverse effects on the overall system performance. The present study compares continuous and intermittent air-gap MD desalination at a lab-scale by evaluating performance parameters and scaling development. Apart from a slightly lower distillate productivity and a similar distillate quality under intermittent conditions, no direct difference in MD performance between continuous and intermittent experiments was detected. Nevertheless, online monitoring by image analysis with optical coherence tomography revealed more advanced scaling development during intermittent operation, with larger scaling volumes and cover ratios, particularly after implementing a membrane rinsing and preservation protocol with demineralized water. Membrane autopsies revealed that intermittency led to alterations in the development of the crystal morphology of predominantly CaCO_3_ scaling. These changes were attributed to enhanced nucleation and modified growth kinetics triggered by recurring shutdown and start-up phases. Overall, the findings showed that intermittency had an adverse effect in terms of scaling behavior, highlighting the need for operating protocols tailored to each specific MD application.

## 1. Introduction for MD and Renewable Energy Systems

Membrane distillation (MD) is a recent separation technology that combines thermal- and membrane-based processes to treat water solutions. MD consists of a hydrophobic porous membrane that separates two channels at different temperatures. The temperature difference between both channels creates a partial vapor pressure difference and thus the driving force that promotes the transport of water vapor from the warm feed water channel (i.e., evaporator) through the membrane. Water vapor then condenses and gets collected as product in the opposite side of the membrane. In principle, this process lets water vapor form, along with allowing volatile substances to pass the membrane, leaving other less-volatile components such as inorganic compounds behind. For this reason, MD is commonly used for desalination applications where high salt rejections are entailed [1,2,3].

MD can be operated at relatively low temperatures and provides flexible modularity to cover small- to medium-scale capacities as opposed to other thermal desalination technologies [4]. In addition, MD has been proven at pilot-scale to be a cost-effective and robust decentralized solution under fluctuating thermal energy sources and operations [5,6,7]. These aspects have allowed the development of MD in several niche applications, especially where low-grade waste heat is available—e.g., in solar plants [8,9], geothermal plants [10] or industrial settings such as in water electrolysis systems [11,12,13].

In membrane distillation, as in other membrane-based desalination techniques, fouling is an important issue that leads to performance deterioration [1,14,15,16,17]. Fouling has been well documented for MD predominantly as inorganic scaling. This phenomenon consists of the precipitation of salts on the membrane surface as a result of concentration and temperature polarization at the membrane–solute interface [15,18]. Scaling is thus highly dependent on the interaction of surface energies between the membrane surface, scaling crystals and water [17,19]. Fouling and scaling lead to the blocking of membrane pores for water to pass through the membrane, compromising membrane productivity [3,15]. Scaling deposition can also lower the hydrophobic properties of the membrane and allow feed solution to enter the membrane pores (i.e., pore wetting) [18]. Different operating conditions such as feed water quality, temperature, flow rates, recovery and pretreatment methods [10,15,20] play an important role in the degree and development of scaling.

In addition, operating modes and module preservation protocols have been shown to exert a significant impact on membrane fouling. Periods of temporarily low water demand or limited (waste) energy supply for desalination or from adjacent systems (e.g., water electrolyzers) can result in partial operation or complete plant shutdowns. In such cases, appropriate measures must be considered to ensure stable MD performance.

Previous research suggests that an intermittent desalination operation may lead to fouling that differs from, or is more severe than, that observed during continuous operations. Moreover, the protocols applied during shutdowns can also affect both fouling and, ultimately, membrane hydrophobicity. Guillen-Burrieza et al. [21] addressed scaling in a pilot-plant desalination study by regularly carrying out cleaning procedures. They reported that long periods of inactivity where the membrane remained in contact with the feed water reversed the cleaning effect and worsened scaling and wetting formation. Additionally, they pointed out the importance of proper shutdown and membrane storage protocols. A recent study by Kim et al. [22] compared different intermittent protocols in a lab-scale direct contact MD system with real seawater. The authors implemented an optimal approach by draining and flushing with clean water followed by draining back the membrane. This protocol provided the best performance regarding scaling, flux and distillate quality compared to only draining and not draining the system. Moreover, the study reported no significant difference between the proposed protocol and continuous operation based on these criteria. Draining and a complete dry-out has been reported by several studies to lead to severe scaling, wetting and low distillate quality [5]. Two different studies [23,24] reported a deterioration of membrane hydrophobicity and physical properties from crystal formation after letting the membranes dry-out during intermittent operation with real brackish water and seawater. Furthermore, Hejazi et al. [25] compared different protocols and found that keeping the feed and permeate solutions in the module during shutdowns helped preserve the membrane and prevent deterioration of salt rejection and the overall performance. This approach was beneficial as opposed to draining out the membranes both with and without a prior demineralized rinsing step. Another study [26] followed similar protocols in a pilot study and reported a better distillate quality in the beginning of each experiment when rinsing and storing the membrane modules with demineralized water. Conversely, a single study [27] stated that there was no significant impact from dry-out protocols after long-term on/off operation with NaCl solutions.

The available literature does not seem to point toward a single best-practice approach to deal with intermittency and its potential effects. It suggests, however, that some sort of maintenance practice should be implemented and the best protocol may vary depending on the operating conditions. Additionally, only a few studies looked into continuous and intermittent operation under comparable conditions. Apart from distillate quality deterioration, the effect of intermittent operation on scaling and crystal formation is not entirely clear.

This study investigates membrane distillation under continuous and intermittent operation on a lab-scale. The work was carried out to compare the long-term effect of operating modes and proposed protocols on membrane performance and fouling. For the analysis, optical coherence tomography as a supporting tool for real-time scaling monitoring was implemented. This work considers a conceptual application of MD desalination for pure-water production for offshore water electrolysis and intends to provide potential protocols for such applications.

## 2. Materials and Methods

### 2.1. Feed Water

Synthetic seawater solutions, simulating North Sea water composition, were prepared by diluting a high salinity thermal water from Bad Wimpfen, Germany, with tap water (Karlsruhe, Germany). Concentrations of magnesium, sulfate and calcium were adjusted by supplementing the solution with MgSO_4_∙7H_2_O (≥98%, VWR International, Radnor, PA, USA) and CaCl_2_∙2H_2_O (≥99.5%, VWR International). The concentrations of major ions of the feed solution measured by inductively coupled plasma-optical emission spectroscopy (ICP-OES 5110, Agilent Technologies, Santa Clara, CA, USA) and ion chromatography (790 Personal, Metrohm, Hersisau, Switzerland) are listed in Table 1. Additionally, 20 mg·L^−1^ of sodium azide (≥99.0%, Merck GmbH, Darmstadt, Germany) was added to the solutions to prevent biological growth during the experiments. Every two to three days of operation, half of the solution (40 L) was replaced by a new batch to maintain bicarbonate concentrations.

### 2.2. MD Setup

**Figure 1:**
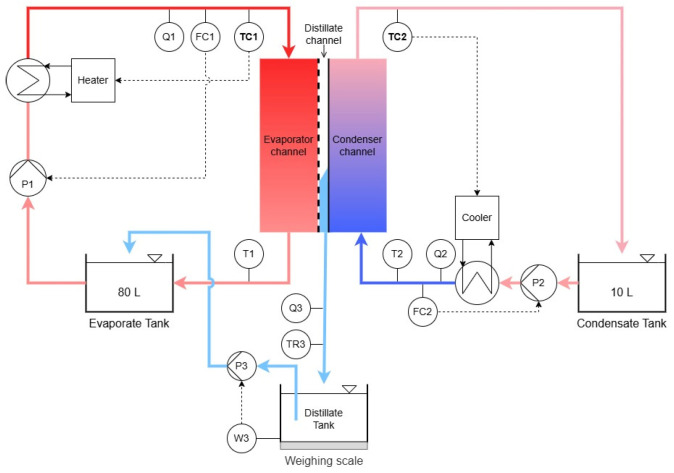
Schematic of AGMD lab setup. The flat-sheet MD module is depicted in the center of the schematic. Sensor and controller (C) abbreviations: T = temperature, F = flow, Q = conductivity, W = weight.

The experiments were performed in an automated laboratory scale air-gap MD setup (SolarSpring GmbH, Freiburg, Germany). A schematic of the setup is shown in Figure 1. The setup consists of an evaporator (feed seawater) and a condenser loop that flow through a flat-sheet MD module. Both the evaporator and condenser streams are recirculated to their corresponding tanks (80 L and 10 L, respectively). Temperature and flow rates in the two separate loops are controlled automatically. During the experiments, the temperature difference across the membrane was adjusted based on the temperatures at the inlet of the evaporator (TC1) and the outlet of the condenser (TC2) shown in Figure 1. Produced distillate is directed from the air gap to a separate line, in which electrical conductivity (Q3) and temperature (T3) are recorded continuously and collected in a tank placed on a scale. To ensure constant salinities in the evaporator loop, distillate is periodically pumped back to the evaporator tank. A list of sensors and components relevant to the laboratory setup including the name of the component and its respective accuracy is provided in the Supplementary Materials.

The flat-sheet MD module was custom-built with a feed plate from polyoxymethylene modified with three optical windows on the evaporator side-plate for image acquisition of the membrane surface. The distances from the feed inlet to the monitoring positions were 5, 13 and 21 cm. For all experiments, pristine membranes of expanded PTFE (0.2 µm, Donaldson 6502, Leuven, Belgium) with an active area of 387.5 cm^2^ were used.

### 2.3. Experimental Conditions

The experiments were conducted at the same conditions in terms of flow rate (100 L·h^−1^) and temperature levels. The top temperature in the evaporator inlet was set at 60 °C to simulate the high-temperature end of a theoretical MD module coupled with waste heat from an electrolysis cell based on Schwantes et al. [11]. Accordingly, a temperature difference between the channels of 15 °C was maintained between the evaporator inlet and condenser outlet.

Three operating modes were performed once as described in Table 2 to simulate continuous and intermittent operations. Under intermittent conditions, an on/off operation was followed in which both pumping and temperature controls were turned off during shutdowns. The intermittency was carried out during week days and the setup was left running on weekends. In one of the intermittent operations, a flushing, rinsing and preservation protocol with demineralized water was implemented during the shutdown phases. In the other intermittent experiment, no specific shutdown protocol was carried out, and the experiment is hereon referred to as “intermittent”. All experiments were conducted for approx. 260 h (11 days) of operation, excluding shutdown time for the case of the intermittent experiments.

### 2.4. Experimental Analysis

#### 2.4.1. Optical Coherence Tomography and Image Processing

An optical coherence tomography (OCT) system from Thorlabs GmbH, Lübeck, Germany, (GANYMEDE-II spectral domain) equipped with an LSM04 objective lens (Thorlabs GmbH, Lübeck, Germany) was used for imaging. Acquisition of three-dimensional datasets were collected at several operating hours. All datasets were taken with a field of view of 8.5 mm × 3.2 mm × 1.5 mm at a pixel resolution of 8 µm × 8 µm × 3.14 µm (x, y and z-axes, respectively). The digital image processing was executed in Python 3 based on the methodology described by Aliaskari et al. [28] by cropping a region of interest inside the membrane spacer and directly above the membrane surface, binarization and flattening of the two-dimensional sets.

For the quantification and evaluation of the scaling layer with OCT data, two parameters were analyzed. The first parameter was defined to describe the percentage of total membrane area $A_{m}$ covered by scaling layer $A_{s}$:

(1) $$Cover\;ratio\;\left[\%\right]=\;\frac{A_{s}}{A_{m}}$$

where $A_{s}$ represents the total count of white pixels on the x-y plane of the binarized dataset and $A_{m}$ the total amount of pixels in the analyzed region of interest.

The location of the highest white pixel on the z-axis was identified and multiplied by the pixel z dimension to generate height maps and estimate the mean height of the scaling layer for each one of the images. The second parameter, scaling volume, was then calculated by multiplying the mean height by $A_{s}$ and the pixel x and y dimensions described above.

#### 2.4.2. Membrane Autopsies

After each experiment, the membranes were taken out of the MD module, carefully cleaned with milli-Q-grade water to remove remaining feed water from the top of the membrane or scaling layer, stored and dried at room temperature. A total of four coupons of 4 × 3 cm per membrane sample were carefully cut and dried at 105 °C for over 24 h. The coupons were then submerged in a 20 mL 0.1 M HCl solution and sonicated for 1 h to dissolve the scaling layer. Subsequently, the liquid phase was diluted accordingly with demineralized water to meet the measuring ranges in the further element analysis.

The element concentrations of the fouling layer were measured by ICP-OES. Based on the sample volume, the total mass of each element was estimated per m^2^ of analyzed membrane area.

Additional coupons were carefully sampled from the middle section of the membranes and dried at room temperature to later examine them with scanning electron microscopy (FEI Quanta FEG 650, FEI, Hillsboro, OR, USA) and energy-dispersive x-ray spectroscopy analysis (Quantax Esprit 1.9 Systems, Bruker, Billerica, MA, USA).

## 3. Results and Discussion

### 3.1. Performance Parameters

**Figure 2:**
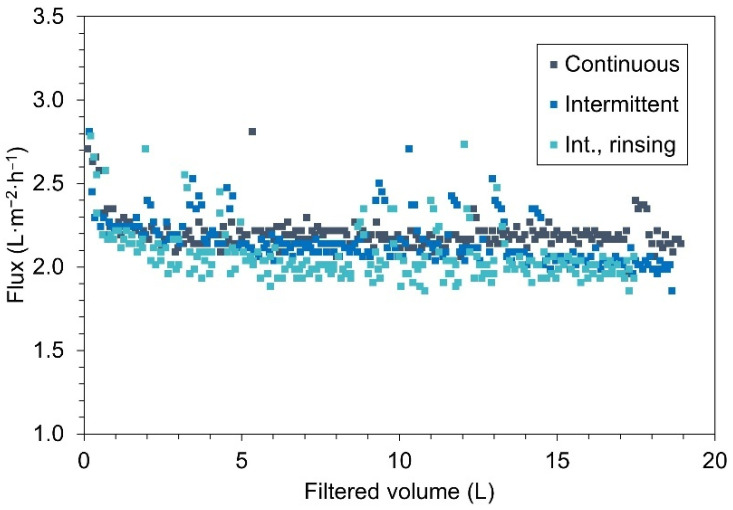
Comparison of flux over cumulative distillate volume for continuous and intermittent (int.) operations.

The recorded flux and distillate conductivity were evaluated as performance parameters of the MD setup. Figure 2 depicts the hourly flux over the filtered volume of distillate for the three experiments. The observed flux values showed to be steady throughout all the experiments from 1.8 to 2.25 L·m^−2^·h^−1^. In all cases, an initial period of around 5 h was required to reach stable flux conditions. Later during operation, fluctuations were observed during restarts (i.e., after shutdown phases) in the intermittent experiments. Whereas, more constant values were recorded during the longest operating periods over weekends at about 5 to 8.5 L and 14 to 19 L of filtered volume. During these longer periods a slight difference between the two intermittent and the continuous operations is visible. These lower fluxes are also clear when comparing total filtered volume after the 260 h run time. Both intermittent operations presented with slightly lower productivity than the continuous experiment, thereby intermittency with rinsing protocols showed the lowest filtered volume.

A relevant deterioration of membrane separation behavior could not be distinguished. This was indicated by the recorded distillate quality. All electrical conductivity values remained between 3 and 10 µS·cm^−1^ throughout most of the operation, indicating the absence of significant membrane wetting. Sudden increases in conductivity of about 5 µS·cm^−1^ were detected directly after restarts in the intermittent operating modes (see the exemplary plot in Figure S1 in Supplementary Materials). The conductivities prior to the shutdowns were recovered within five hours after restarting operation. A similar phenomenon has been reported in previous studies [21,25,26], where sudden increases in both distillate production and conductivity were observed after restarting the MD desalination systems. However, the documented spikes in conductivity in those studies were significantly larger (i.e., from 200 to higher than 33,000 µS·cm^−1^) and were mainly attributed to leaks at defective membrane spots or to the flushing of salts that had crystallized during shutdown after draining the membrane. In contrast, the changes in the present study are rather low (5 µS·cm^−1^) and therefore not likely to be clarified by these mechanisms. Instead, such minimal variations might have resulted from changes in temperatures in the distillate line (Figure S2, Supplementary Materials), together with slight increases in dissolved carbon dioxide in the distillate, resulting from higher CO_2_ concentrations when feed water cooled down during shutdowns. These explanations are also conceivable when a new batch of seawater was introduced, which was followed by a similar increase in conductivity (Figure S1, Supplementary Materials). Nevertheless, these general parameters do not hint to a pronounced decline in performance attributed to the implementation of intermittent operation.

### 3.2. Scaling Evaluation

**Figure 3:**
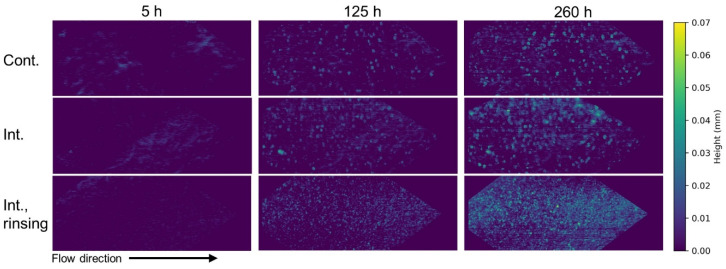
Height maps of scaling layer at different operating times (Int. means intermittent operation).

The observation of the membrane surface by OCT during operation demonstrated accumulation of a scaling layer in all experiments. Figure 3 shows the height maps of the scaling layer for exemplary OCT images from the three experiments at different operating times. At first glance, different scaling structures are evident in terms of spatial distribution, particularly in the intermittent operation with rinsing protocol. A quantitative evaluation and comparison are discussed in the following sections.

#### 3.2.1. Scaling Development

**Figure 4:**
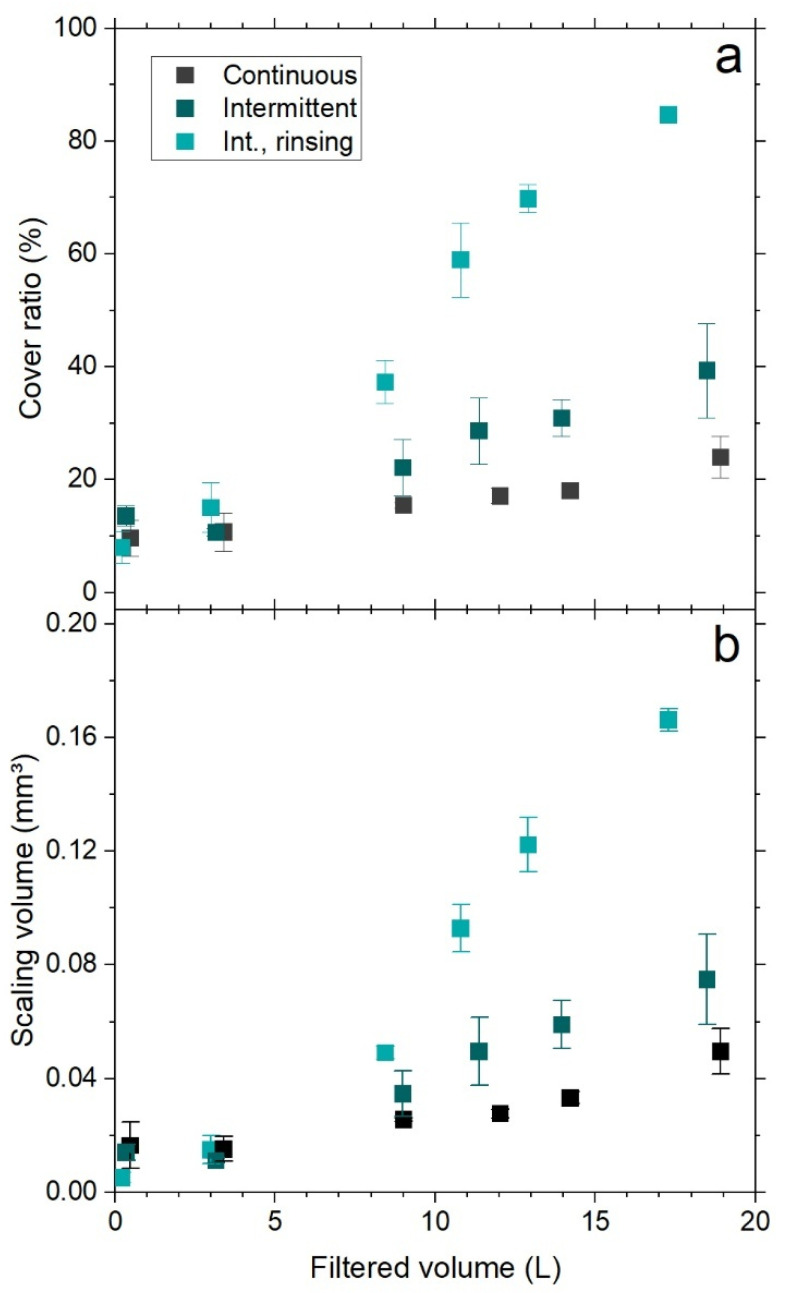
(**a**) Cover ratio and (**b**) scaling volume over operating time for the different operations (Int. means intermittent operation).

Figure 4a,b shows the cover area and scaling layer volume for the three experiments. The plotted data represent the mean value and standard deviations of all three monitoring positions at the given operating time.

As seen in Figure 4a, cover ratios (i.e., percentage of membrane area covered by scaling) in all experiments increased steadily over operating time. Due to the applied image processing method and curvature of the membrane material, a small portion of the membrane surface is always detected and quantified as the scaling layer. This explains the starting cover ratios of about 10% in the first hours of operation. After about 125 h, the highest values were estimated for the intermittent operation with rinsing. In this case, the cover ratio continuously increased and scaling covered most of the membrane area by the end of the experiment (approx. 80%). The lowest ratios were observed during the continuous conditions, followed by the intermittent experiment. These differences are also directly visible in the height maps (Figure 3).

A similar trend compared to the cover ratio was observed for scaling volume. The highest volumes were calculated for the intermittent operation with rinsing followed by intermittent and continuous operation. Coincidently, the OCT image evaluation showed that comparable mean values for scaling height between 0.01 and 0.015 mm were reached among all three experimental conditions. This explains why the increases in scaling volume were closely proportional to their corresponding cover ratio profiles.

Based on the quantification and height maps, there is a clear variation in scaling morphology and distribution. The intermittent and continuous operations showed some similarities in terms of the crystal structure on a macroscale (i.e., at OCT resolution), characterized by a combination of punctual and high tower-like structures surrounded by a lower scaling layer.

Both the cover ratio and scaling volume hint to an effect of the intermittent operation in which these lower structures are more predominant, likely consisting of new nucleation sites due to more advanced scaling development. This can be attributed to the difference in thermodynamic conditions between both operating modes. In the continuous operation, feed water was maintained at a constant temperature throughout the experiment. In the intermittent operation, the membrane and the scaling layer were exposed to reductions to room temperatures (20–25 °C) during shutdowns. More importantly, restarts led to rapid increases in temperature, likely creating sudden supersaturation of sparingly inorganic salts. Highly supersaturated states resulted thus in favored nucleation [29,30].

Smaller crystal structures in the intermittent operation with rinsing may be attributed to the controlled growth by preserving the membrane module with demineralized water or rather the reduction in contact time between crystals and the seawater solution. It has been previously reported that crystal growth is mainly mediated by the supersaturated bulk solution and can be controlled independently from crystal nucleation [29]. Apart from the degree of supersaturation, the supersaturation rate is also presumed to play an important role in nucleation kinetics. Increases in the rate at which supersaturation in the crystal/solution interface is exceeded can lead to more predominant nucleation rates and smaller crystal sizes [31,32,33]. It may be therefore hypothesized that increased supersaturation rates in the transition from demineralized water and feed seawater right after restart likely enhanced nucleation when introducing rinsing protocols, explaining the larger scaling volumes and cover ratios.

The more predominant scaling development during intermittent operation can be linked to the lower distillate productivities recorded in these operations (Figure 2). Although both intermittent experiments exhibited similarly minor flux declines, previous studies suggest that flux behavior is largely independent of crystal morphology and surface coverage [19].

#### 3.2.2. Scaling Morphology

**Figure 5:**
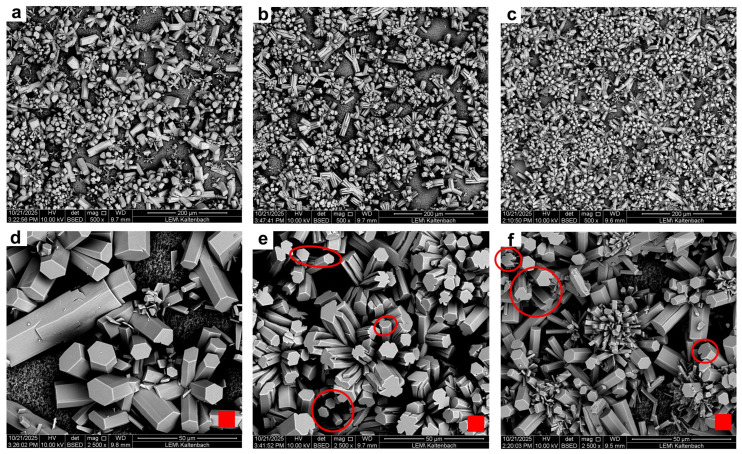
SEM images of scaling layer by the end of the 260 h experiments under (**a**,**d**) continuous, (**c**,**f**) intermittent without rinsing and (**b**,**e**) intermittent with rinsing operations. Circles in (**f**) show exemplary small crystal structures without alteration of hexagonal faces. Circles in (**e**) show exemplary crystals with altered amorphous faces. The red squares in (**d**–**e**) depict the OCT pixel resolution in the x-y plane (8 × 8 µm).

SEM analysis confirmed the difference in scaling development as well as differences in crystal morphology. As observed in Figure 5a–e, the scaling layer for the operation with rinsing appeared to cover most of the membrane area, while larger portions of free membrane were visible in the background for the other two operations.

For the continuous conditions, the crystals exhibited large, well-defined hexagonal prism-shaped structures. In the intermittent operation without rinsing, the crystal structures showed a significant difference in morphology. Intermittency produced amorphous lateral faces in the single structures. A trend is visible in which the smallest crystals show hexagonal prismatic morphologies similar to continuous operation (circled in Figure 5f). As the crystals grew in size, these tended to more amorphous structures. Aside from potentially increasing nucleation, intermittency appeared to also modify growth patterns of the single crystals.

A different phenomenon was detected when the rinsing shutdown protocol was introduced. The analyzed samples depicted an increase in the number as well as more variation in crystal structures. Hexagonal prismatic morphologies were observed, yet in a smaller size distribution than in the other experiments. As previously hypothesized, sudden supersaturation states may induce new nucleation sites, whereas rinsing and preserving the membrane with demineralized water may also limit the growth of existing crystals. Secondly, conglomerations of small plate-like crystals were distinguishable, indicating a shape transformation. A previous study [34] reported the impact of sudden exposure of calcium carbonate crystals to demineralized water. A dissolution–recrystallization effect was suggested to explain a complete shift of crystal phases. The effect was more significant the longer the exposure. Moreover, indications of a similar trend toward amorphous structures were also present, particularly in the larger formations marked in Figure 5e. In this operating mode, however, the effect appears less pronounced, possibly due to the controlled growth induced by the shutdown protocol.

#### 3.2.3. Scaling Composition

**Figure 6:**
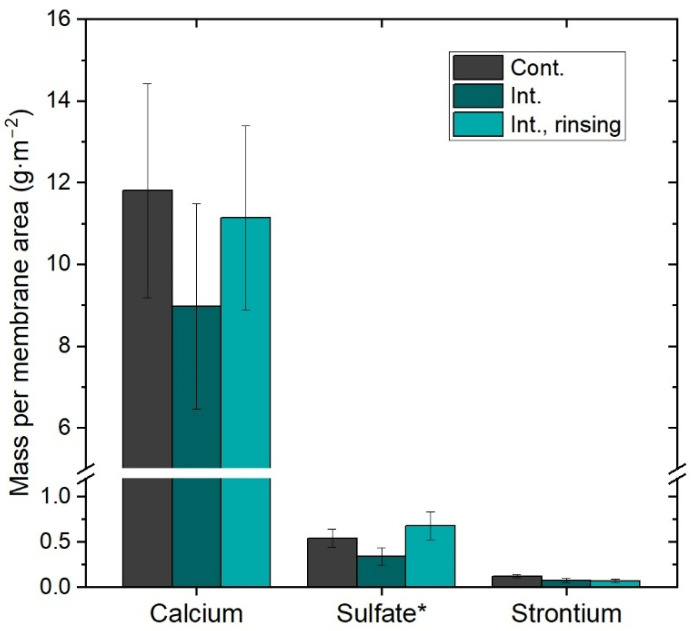
Measured mass of predominant inorganic species in scaling layer per membrane area. * Sulfate was estimated based on the measured sulfur concentration (Int. means intermittent operation).

The ex situ membrane autopsies performed after operation validated the presence of inorganic scaling. Figure 6 presents the measured mass of the inorganic species per analyzed membrane area. Calcium and sulfate followed by strontium were the major constituents detected in all experiments. In contrast, magnesium and barium were measured at rather negligible values and are therefore excluded in the graph.

An important difference in scaling layer composition among the operating modes was not noticeable. The results hint toward scaling layers of carbonate species (e.g., CaCO_3_, SrCO_3_). EDX analysis confirmed the presence of mostly calcium in all three experiments, indicating that the scaling layer consisted of different morphologies of calcium carbonate crystals. This finding is supported by previous research demonstrating that CaCO_3_ can exhibit a wide range of crystal structures depending on concentration (i.e., saturation state), temperature profile, as well as separation process [35,36]. Moreover, the scaling composition in this study was essentially independent of the operating conditions.

The measured masses per membrane area were comparable among all three operations, despite the OCT analysis estimating larger cover ratios and scaling volumes for the intermittent operations. This difference can be explained by two factors: (1) the autopsy methodology, which may not ensure a complete dissolution of the scaling layer and instead provides qualitative information on composition, and (2) the constrains of the imaging method and resolution. The detection of fouling layers through OCT largely depends on the pixel resolution of the device. Thus, any voids or empty spaces between crystal structures smaller than the pixel size may be interpreted as solid scaling material, resulting in overestimated volumes and cover ratios. This effect is presumably more present in morphologies with conglomerations of small crystals—such as in the intermittent operation with rinsing—compared to morphologies with large, well-defined structures as observed under continuous operation. This is illustrated by comparing the actual pixel size with the crystal size and morphology at high magnifications (see Figure 5d,e). Consequently, OCT is not able to provide information about scaling porosity or density, which limits the direct comparison of OCT parameters with scaling mass.

Nevertheless, the results suggest an evident effect of intermittency on both distillate productivity and the development of the scaling layer and crystal morphology. This may be attributed to the alteration of the supersaturation state and its rate of increase by sudden changes in concentration and temperature, which are known to shift the morphology of inorganic crystals [29,31,32]. Additionally, introducing a rinsing protocol did not appear to be a beneficial approach for the studied water and operating conditions, as reported in previous studies [22,26]. Therefore, the findings further indicate the need for application-specific measures and optimized protocols to address intermittency and limit its effect on MD performance.

## 4. Conclusions

In this study, continuous and intermittent operation of air-gap MD desalination was investigated on a lab-scale to evaluate performance parameters and scaling behavior, as well as to assess the impact of intermittency on these criteria. Experimental conditions were selected to simulate a conceptual application of MD coupled with offshore water electrolysis. During the intermittent operation (i.e., on/off operation), two experiments were conducted, with and without implementation of a rinsing and preservation protocol with demineralized water.

The results showed that distillate flux was slightly lower under intermittent conditions, whereas distillate quality remained comparable across all experiments, with slight increases (<5 µS·cm^−1^) detected after restarting operation. The effect of intermittency was more evident in terms of scaling development. OCT imaging suggested a more advanced scaling under intermittent operation. The largest scaling volumes and cover ratios were quantified under intermittent conditions, especially when applying a rinsing protocol. Although this observation is consistent with the enhanced nucleation kinetics associated with supersaturation states driven by shutdown and start-up phases, possible overestimations associated with OCT as a quantification tool must be considered. These limitations arise from its inability to detect small-enough (i.e., smaller than the pixel resolution) voids between crystal structures as well as to provide information on scaling density. Nevertheless, the impact of intermittency on scaling development aligns well with the reduced distillate productivity.

Furthermore, membrane autopsies revealed the presence of predominantly CaCO_3_ crystals with substantial morphology variations among the different operating conditions. While continuous operation led to well-defined hexagonal prismatic structures, intermittent operation without rinsing resulted in altered crystal morphology, characterized by the appearance of a larger number of smaller crystals with amorphous structures. These differences were even more distinguishable after a rinsing protocol was implemented, resulting in a more drastic nucleation and shape variations. It was therefore hypothesized that shutdown and start-up phases, together with the preservation with demineralized water, may manipulate both nucleation and growth kinetics by inducing sudden transitions into supersaturation states. This led to altered scaling development compared to continuous operation.

Overall, the findings further confirm that universal protocols for intermittent MD operations are unlikely to be developed and that operating strategies should instead be tailored to the specific application, considering factors such as feed water composition, added chemicals, temperature profiles, membrane material and shutdown durations.

## Figures and Tables

**Table 1:** Average feed water composition. Cation concentrations were analyzed as element.

Parameter		Unit
Cl^−^	19,085	mg·L^−1^
Na	11,344	mg·L^−1^
SO_4_^2−^	2694	mg·L^−1^
Mg	585	mg·L^−1^
Ca	418	mg·L^−1^
HCO_3_^−^	197	mg·L^−1^
Sr	2.6	mg·L^−1^
Ba	0.04	mg·L^−1^
El. Conductivity	49.8	mS·cm^−1^
pH	7.7	-

**Table 2:** Description of operation configurations for MD experiments.

Operating Mode	Intermittency	Shutdown Protocol	Operating Time
Continuous	N.A.	N.A.	~260 h
Intermittent	18 h on/ 6 h off	No rinsing. Leaving feed water inside MD module.	~260 h
Intermittent, rinsing	18 h on/ 6 h off	Brief flush-out followed by rinsing (~10 min at 150 L·h^−1^; room temperature) with demin. water. Leaving demin. water inside MD module.	~260 h

N.A.: not applicable.

## Data Availability

The original contributions presented in this study are included in the article/Supplementary Material. Further inquiries can be directed to the corresponding authors.

## Supplementary Materials

The following supporting information can be downloaded at: https://www.mdpi.com/article/10.3390/membranes16040144/s1. Table S1: List of equipment of the AGMD lab setup; Figure S1: Comparison of distillate conductivity over operating time for the different operations. Reference lines depict restarts. Sudden increase in conductivity in the continuous experiment after 70 h was caused by the introduction of a new feed seawater batch; Figure S2: Comparison of recorded distillate temperature over operating time for the different operations. Reference lines depict restarts.

## References

[1] Alkhudhiri A., Darwish N., Hilal N. (2012). Membrane distillation: A comprehensive review. Desalination.

[2] Tong T., Elimelech M. (2016). The Global Rise of Zero Liquid Discharge for Wastewater Management: Drivers, Technologies, and Future Directions. Environ. Sci. Technol..

[3] Horseman T., Yin Y., Christie K.S.S., Wang Z., Tong T., Lin S. (2021). Wetting, Scaling, and Fouling in Membrane Distillation: State-of-the-Art Insights on Fundamental Mechanisms and Mitigation Strategies. ACS EST Eng..

[4] Deshmukh A., Boo C., Karanikola V., Lin S., Straub A.P., Tong T., Warsinger D.M., Elimelech M. (2018). Membrane distillation at the water-energy nexus: Limits, opportunities, and challenges. Energy Environ. Sci..

[5] Guillén-Burrieza E., Blanco J., Zaragoza G., Alarcón D.-C., Palenzuela P., Ibarra M., Gernjak W. (2011). Experimental analysis of an air gap membrane distillation solar desalination pilot system. J. Membr. Sci..

[6] Zhang H., Xian H. (2024). Review of Hybrid Membrane Distillation Systems. Membranes.

[7] Amaya-Vías D., López-Ramírez J.A. (2019). Techno-Economic Assessment of Air and Water Gap Membrane Distillation for Seawater Desalination under Different Heat Source Scenarios. Water.

[8] Ali E. (2022). Optimal Control of Direct Contact Membrane Distillation Operated under Fluctuating Energy Source. Membranes.

[9] Khayet M. (2013). Solar desalination by membrane distillation: Dispersion in energy consumption analysis and water production costs (a review). Desalination.

[10] Huang F.Y.C., Arning A. (2019). Performance Comparison between Polyvinylidene Fluoride and Polytetrafluoroethylene Hollow Fiber Membranes for Direct Contact Membrane Distillation. Membranes.

[11] Schwantes R., Morales Y., Pomp E., Singer J., Chavan K., Saravia F. (2025). Thermally driven ultrapure water production for water electrolysis—A techno-economic analysis of membrane distillation. Desalination.

[12] Arthur T., Millar G.J., Love J. (2023). Integration of waste heat recovered from water electrolysis to desalinate feedwater with membrane distillation. J. Water Process Eng..

[13] Arthur T., Millar G.J., Sauret E., Love J. (2023). Renewable hydrogen production using non-potable water: Thermal integration of membrane distillation and water electrolysis stack. Appl. Energy.

[14] Chen L., Xu P., Wang H. (2020). Interplay of the Factors Affecting Water Flux and Salt Rejection in Membrane Distillation: A State-of-the-Art Critical Review. Water.

[15] Tong T., Wallace A.F., Zhao S., Wang Z. (2019). Mineral scaling in membrane desalination: Mechanisms, mitigation strategies, and feasibility of scaling-resistant membranes. J. Membr. Sci..

[16] Gryta M. (2008). Fouling in direct contact membrane distillation process. J. Membr. Sci..

[17] Rolf J., Cao T., Huang X., Boo C., Li Q., Elimelech M. (2022). Inorganic Scaling in Membrane Desalination: Models, Mechanisms, and Characterization Methods. Environ. Sci. Technol..

[18] He F., Gilron J., Lee H., Song L., Sirkar K.K. (2008). Potential for scaling by sparingly soluble salts in crossflow DCMD. J. Membr. Sci..

[19] Bauer A., Wagner M., Horn H., Saravia F. (2021). Operation conditions affecting scale formation in membrane distillation—An in situ scale study based on optical coherence tomography. J. Membr. Sci..

[20] Abdel-Karim A., Leaper S., Skuse C., Zaragoza G., Gryta M., Gorgojo P. (2021). Membrane cleaning and pretreatments in membrane distillation—A review. Chem. Eng. J..

[21] Guillen-Burrieza E., Ruiz-Aguirre A., Zaragoza G., Arafat H.A. (2014). Membrane fouling and cleaning in long term plant-scale membrane distillation operations. J. Membr. Sci..

[22] Kim H.-W., Jang A., Jeong S. (2023). Operational strategy preventing scaling and wetting in an intermittent membrane distillation process. npj Clean Water.

[23] Guillen-Burrieza E., Thomas R., Mansoor B., Johnson D., Hilal N., Arafat H. (2013). Effect of dry-out on the fouling of PVDF and PTFE membranes under conditions simulating intermittent seawater membrane distillation (SWMD). J. Membr. Sci..

[24] Wang S., You Y., Wang X., Huang W., Zheng L., Li F. (2023). Fouling mechanism and effective cleaning strategies for vacuum membrane distillation in brackish water treatment. Desalination.

[25] Hejazi M.-A.A., Bamaga O.A., Al-Beirutty M.H., Gzara L., Abulkhair H. (2019). Effect of intermittent operation on performance of a solar-powered membrane distillation system. Sep. Purif. Technol..

[26] Ruiz-Aguirre A., Andrés-Mañas J.A., Zaragoza G. (2019). Evaluation of Permeate Quality in Pilot Scale Membrane Distillation Systems. Membranes.

[27] Wang Y., Xu Z., Lior N., Zeng H. (2015). An experimental study of solar thermal vacuum membrane distillation desalination. Desalination Water Treat..

[28] Aliaskari M., Horn H., Saravia F. (2025). Real time monitoring of scaling behavior in bipolar membrane electrodialysis. J. Membr. Sci..

[29] Jikazana A., Campo P., McAdam E.J. (2023). Hydrodynamics (Reynolds number) determine scaling, nucleation and crystal growth kinetics in membrane distillation crystallisation. J. Membr. Sci..

[30] Edwie F., Chung T.-S. (2013). Development of simultaneous membrane distillation–crystallization (SMDC) technology for treatment of saturated brine. Chem. Eng. Sci..

[31] Aquilano D., Pastero L., Bruno M., Rubbo M. (2009). {100} and {111} forms of the NaCl crystals coexisting in growth from pure aqueous solution. J. Cryst. Growth.

[32] Vasilakos K., Thomas N., Hermassi M., Campo P., McAdam E. (2025). On the role of crystal-liquid interfacial energy in determining scaling, nucleation and crystal growth in membrane distillation crystallisation. J. Membr. Sci..

[33] Kim S., Jeon J., Kim M.-J. (2022). Vaterite production and particle size and shape control using seawater as an indirect carbonation solvent. J. Environ. Chem. Eng..

[34] Sarkar A., Mahapatra S. (2010). Synthesis of All Crystalline Phases of Anhydrous Calcium Carbonate. Cryst. Growth Des..

[35] Ma M., Wang Y., Cao X., Lu W., Guo Y. (2019). Temperature and Supersaturation as Key Parameters Controlling the Spontaneous Precipitation of Calcium Carbonate with Distinct Physicochemical Properties from Pure Aqueous Solutions. Cryst. Growth Des..

[36] Zhang Z., Wadekar S.S., Lokare O.R., Vidic R.D. (2021). Comparison of calcium scaling in direct contact membrane distillation (DCMD) and nanofiltration (NF). J. Membr. Sci..

