# Supplementary material for: Understanding Scaling Development in Intermittent MD Operation
Source: Membranes (Basel). 2026 Apr 9;16(4):144. doi: 10.3390/membranes16040144 (PMC13117879; doi:10.3390/membranes16040144)
Supplement: Supplementary file 1 [file membranes-16-00144-s001.zip › membranes-4212598-supplementary.pdf]

Supplementary Materials

Understanding Scaling Development in Intermittent MD Operation

Yair Morales [1,*], Jan Singer [1], Leonardo Acero [2], Harald Horn [1,2,*] and Florencia Saravia [1]

[1] DVGW-Research Center at the Engler-Bunte-Institut, Water Chemistry and Water Technology, Karlsruhe Institute of Technology, Engler-Bunte-Ring 9, 76131 Karlsruhe, Germany;

[2] Karlsruhe Institute of Technology, Engler-Bunte-Institut, Water Chemistry and Water Technology,
Engler-Bunte-Ring 9, 76131 Karlsruhe, Germany;

* Correspondence: morales@dvgw-ebi.de (Y.M.); harald.horn@kit.edu (H.H.)

Table S1. List of equipment of the AGMD lab setup.

Component	Producer	Type	Accuracy	Label
Conductivity meters	Bürkert	8228	± (0.002 × measured value + 5 µS·cm^{-1})	Q1, Q2
Conductivity meter di stillate	Jumo	BlackLine CR-EC, K=0.1	±2%; 0.1 □s·cm^{-1} - 1 mS·cm^{-1}	Q3
Volume flow meter	Krohne	OPTIFLUX 4000	0.3% of measured value	FC1, FC2
Temperature sensors	TC direct	Pt100	± (0.15+0.002 × T)	TC1, TC2, T1, T2, T3
Pumps	Pentair	Shurflo 2088		P1, P2
Pump distillate	Boxer	3MD		P3
Balance distillate	Soehnle	Table balance	± 1 g; 0 – 32 kg	W3
Cooler	Huber	Unichiller 025-H	±2%	Cooler

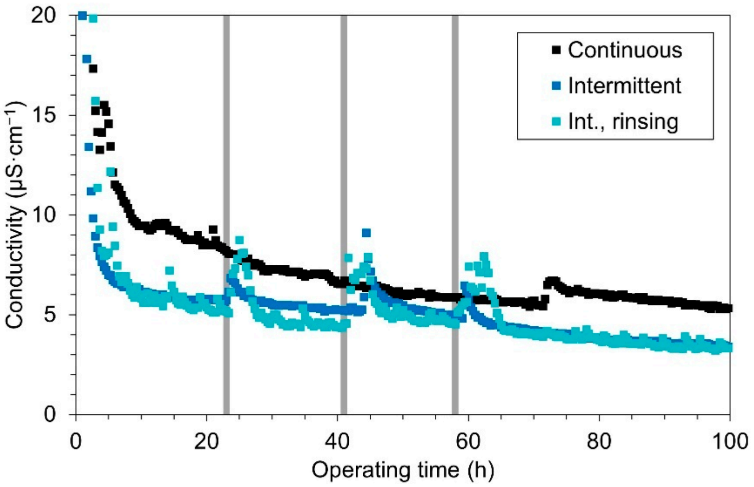

Figure S1. Comparison of distillate conductivity over operating time for the different operations. Reference lines depict restarts. Sudden increase in conductivity in the continuous experiment after 70 h was caused by the introduction of a new feed seawater batch.

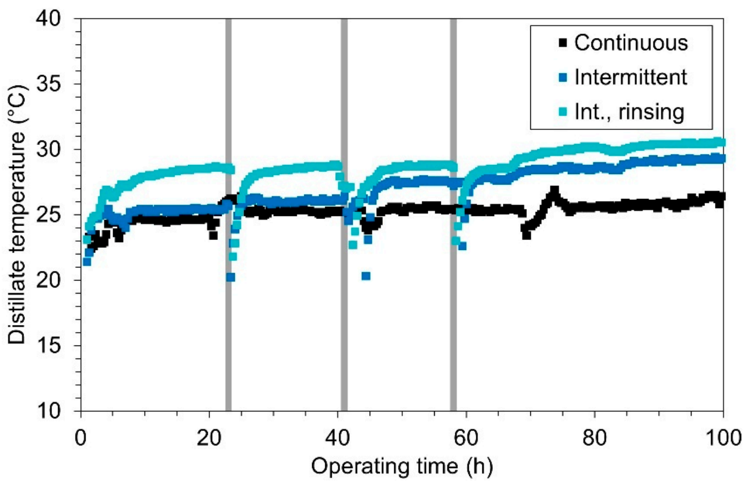

Figure S2. Comparison of recorded distillate temperature over operating time for the different operations. Reference lines depict restarts.
